# Disease acceptance and adherence to imatinib in Taiwanese chronic myeloid leukaemia outpatients

**DOI:** 10.1007/s11096-013-9867-8

**Published:** 2013-10-24

**Authors:** Li-Chia Chen, Teng-Chou Chen, Yaw-Bin Huang, Chao-Sung Chang

**Affiliations:** 1 Division for Social Research in Medicines and Health, School of Pharmacy, University of Nottingham, East Drive, University Park, Nottingham, NG7 2RD UK; 2 Graduate Institute of Clinical Pharmacy, Kaohsiung Medical University, Kaohsiung, Taiwan; 3 Division of Hematology-Oncology, Department of Internal Medicine, Kaohsiung Medical University Hospital, Kaohsiung Medical University, Kaohsiung, Taiwan

**Keywords:** Acceptance, Adherence, Adverse drug reaction, Chronic myeloid leukaemia, Polypharmacy, Taiwan

## Abstract

*Background* The launch of imatinib has turned chronic myeloid leukaemia (CML) into a chronic illness due to the dramatic improvement in survival. Several recent studies have demonstrated that poor adherence to imatinib may hamper the therapeutic outcomes and result in increased medical expenditures, whilst research on exploring the reasons for non-adherence to imatinib is still limited. *Objective* This study aimed to explore the experience of patients as they journey through their CML treatments and associated imatinib utilisation in order to understand the perceptions, attitudes and concerns that may influence adherence to imatinib treatment. *Setting* This study was conducted at oncology outpatient clinics in a medical centre in southern Taiwan. *Methods* CML patients who regularly attended the oncology outpatient clinics to receive imatinib treatment from October 2011 to March 2012 were invited to participate in the study. Semi-structured face-to-face interviews were used to explore patients’ experiences and views of their treatment, their current CML status and CML-related health conditions, their concerns about imatinib treatment and imatinib-taking behaviours. Patient interviews were recorded, transcribed verbatim and thematically analysed using the constant comparison approach. *Main outcome measure* Themes related to patients’ views of the disease and health conditions, worries and concerns influencing imatinib utilisation behaviours are reported. *Results* Forty-two CML patients participated in the interviews. The emerging themes included: acceptance of current disease and health status, misconceptions about disease progression, factors associated with adherence to imatinib, concerns and management of adverse drug effects. Participants regarded CML as a chronic disease but had misconceptions about disease progression, therapeutic monitoring, resistance to imatinib and symptoms of side effects. Participants were generally adherent to imatinib and favoured long-term prescriptions to avoid regular outpatient visits for medication refills. Experiencing adverse effect was the main reason influencing adherence and led to polypharmacy. Most participants altered medicine-taking behaviours to maintain long-term use of imatinib. *Conclusion* Taiwanese CML patients are adherent to imatinib but report changing their medication-taking behaviour due to adverse drug effects and associated polypharmacy. Patients’ misconceptions of the disease and medication suggests that it is necessary to improve communication between patients and healthcare professionals. Routinely providing updated information as part of the patient counselling process should be considered as a means of improving this communication.

## Impact of findings on practice statements

- Despite being adherent to the treatment, CML patients in Taiwan lack sufficient knowledge of disease progression, therapeutic effects, symptoms and management of side effects.
- Adverse drug effects and associated polypharmacy are the key concerns that impact on patients’ adherence and alter their medicine-taking approach to maintaining long-term use of imatinib.
- Healthcare professionals can help improve adherence and patient care by offering patients information on interpreting clinical indicators, symptoms of adverse effects and strategies to manage adverse effects.

## Introduction

Adherence has been defined by the World Health Organisation as the extent to which a person’s behaviour corresponds with the agreed recommendations of a healthcare provider [1]. Non-adherence to pharmacotherapy has been reported to be associated with increased healthcare costs due to poor health outcomes and a waste of drugs. Non-adherence presents a major problem in health care [2, 3] across different ages and in all therapeutic areas [4, 5]. This is especially true for chronic diseases where the non-adherence rate to long-term therapy is estimated to be 50 % in developed countries [6, 7], and is even higher in developing countries [1]. Non-adherence is also the main reason why patients fail to meet the therapeutic targets for chronic diseases thus resulting in suboptimal health outcomes [8, 9].

With the rapid development of cancer treatments, concerns about the challenges in maintaining adherence (taking medication as prescribed) and persistence (continuing treatment for the prescribed duration) to long-term oral cancer therapies has also been raised [10]. Although the adherence rate for oral cancer therapies is superior to that for oral non-cancer therapies possibly due to the higher motivation of cancer patients and their preference to oral therapies [11, 12], the adherence and persistence rates for oral cancer medications are generally lower in real world settings compared to those in clinical trials, especially for chronically administered medications [13–15]. The average adherence rate for oral anticancer therapies among adults is estimated to be 79 % [16], but it ranges between 0 and 83 % [17] due to different measurements and definitions of adherence.

Since the launch of imatinib, it has transformed chronic myeloid leukaemia (CML) from an inexorably fatal illness to a chronic illness due to its dramatic improvements on survival [18]. Intentional non-adherence to this potentially lifesaving therapy seem counterintuitive, yet several recent studies have demonstrated that non-adherence to imatinib is frequent, and thus may significantly affect the therapeutic outcomes [18, 19] and lead to increased medical expenditures [20]. So far, few studies have explored the factors associated with non-adherence to imatinib therapy in Western countries [21, 22].

The WHO has categorised the determinants of non-adherence to medicines into five dimensions: social and economic, health system-related, therapy-related, condition-related, and patient-related [1]. Previous studies have predominantly evaluated non-cancer chronic conditions and identified several determinants to non-adherence [7]: complexity of therapy, duration of therapy, characteristics of the disease [23], adverse drug reactions [1, 24], cost of treatment [23, 25], characteristics of health service provision, interaction between the prescriber and patient [26], prescribers’ follow-up [27], multiple providers [26] socioeconomic variables [1], multiple medication [26], the patients’ own view of the required therapy [24, 28, 29] and unintended non-adherence [28].

In Taiwan, CML treatment is delivered under the coverage of the Taiwan National Health Insurance (NHI), where imatinib and other second-generation tyrosine kinase inhibitors (TKIs) are available for CML patients who are exempted from co-payment. Similar to other developed countries, since the launch of imatinib in Taiwan in 2003, the survival rate of CML patients has been dramatically improved. However, little is known about Taiwanese CML patients’ adherence to imatinib and the factors that may influence adherence to this treatment regimen.

## Aim of the study

This study explored CML patients’ experiences of treatment processes and use of imatinib, and aimed to understand patients’ perceptions, attitudes and concerns that may influence adherence to imatinib treatment.

## Methods

### Study design and setting

This study adopted a qualitative approach in order to explore patients’ complex medication-taking behaviours and attitudes [30]. Semi-structured face-to-face interviews were conducted from October 2011 to March 2012 at oncology outpatient clinics in a medical centre in southern Taiwan after being granted ethical approval from the Institutional Review Board of the research centre (reference: KMUH-IRB-20110160). The research centre, together with other two medical centres, provides tertiary care for about 3.26 million inhabitants in southern Taiwan, and there are about 6,000 outpatients visiting the research centre daily. At the time of the research, it was estimated that 120 CML patients received treatment there, and about 48 patients were regularly followed up and treated with imatinib.

### Participants

A purposive sample of CML patients who received imatinib treatment was selected as the study population. Eligible patients included those who (1) were diagnosed with CML, (2) regularly visited oncology outpatient clinics to receive imatinib treatment, (3) were able to communicate using either Mandarin or Taiwanese, and (4) without any cognitive impairment. Patients were referred to an onsite research pharmacist (Chen TC) by physicians and invited to participate in this study. The original plan was to interview 40 CML patients visiting the research centre or until theme saturation was reached.

### Data collection

Semi-structured interviews were conducted by a research pharmacist (Chen TC) in a quiet room near the clinic before or after patients’ appointments using a pre-piloted interview schedule which contained open-ended questions about treatment and disease course (“Appendix”). The interview schedule was developed by reviewing published literature [21, 31] and refined after obtaining expert opinion. The pilot study involved three volunteers and three CML patients to ensure the feasibility of the interview schedule, and the pilot results of the CML patients were also included in the data analysis.

Participants were informed of the purpose, interview process and the approximate duration of the interview (30 min). Moreover, consent to participate in the interview and permission to audio-record the interview were obtained. In addition to this, participants’ demographic and socio-economic data were collected using a short questionnaire and the imatinib treatment history was recorded by reviewing individual patient’s medical charts.

### Data analysis

All interviews were recorded, transcribed verbatim and analysed by two researchers (Chen TC and Chen LC) independently using a constant comparison approach until the saturation of emerging themes [32] was achieved and no new issues were identified. Consensus on themes was reached by discussions within the research team.

## Results

### Participants’ characteristics

Overall, 50 CML patients were invited and 42 (aged between 20 and 80 years old) participated in the interviews. Most participants were male (n = 23) with no other co-morbidity (n = 32) and they were diagnosed and treated with imatinib at the chronic phase of CML (n = 35). All participants were receiving imatinib treatment at the time of interview, and 36 participants had been regularly followed for more than 18 months. The majority of participants (n = 36) achieved complete cytogenetic remissions at the 18th month of imatinib treatment, yet some (n = 13) had experienced CML progression to accelerated or blast phase during treatment (Table 1).

**Table 1:** Characteristics of participants

Characteristic	Category	Number (%)
Gender	Male	23 (54.8)
	Female	19 (45.2)
Age^a^	Over 50	22 (52.4)
	Under 50	20 (47.6)
Employment status	Employed	25 (59.5)
	Unemployed	1 (2.4)
	Housekeeper	6 (14.3)
	Retired	9 (21.4)
	Student	1 (2.4)
Marriage status	Single	12 (28.6)
	Married	25 (59.5)
	Widowed	3 (7.1)
	Divorce	2 (4.8)
Use of imatinib for more than 18 months		36 (85.7)
Imatinib utilisation	Prescription interruption^c^	12 (28.6)
	PPR more than 90 %^d^	38 (90.5)
Disease phase at diagnosis	Chronic phase	35 (83.3)
	Accelerated or blast phase	7 (16.7)
Experience of progression^b^		13 (31.0)
Treatment effect	CCyR at the 18th month^e^	36 (90.0)
	MMR at the 18th month^f^	33 (84.6)

^a^Mean age and standard deviation: 50.0 ± 16.0 years

^b^Progression to accelerate or blast phase

^c^Prescription interruption: any gap of prescription covering days between two consecutive imatinib prescriptions for more than 30 days

^d^ *PPR* prescription possession ratio, which refers to the proportion of medication covering days over the treatment period

^e^ *CCyR* complete cytogenetic remissions

^f^ *MMR* major molecular response

The main themes related to participants’ views of the disease and health conditions, worries and concerns influencing imatinib utilisation behaviours including acceptance of current disease and health status, misconceptions about disease progression, factors associated with adherence to imatinib, concerns and management of adverse drug effects.

### Acceptance of current disease and health status

Most participants perceived CML as a severe type of cancer which is easy to progress or metastasise and difficult to treat. Having received imatinib treatment, most participants accepted the reality, were satisfied with their current health status and considered their health conditions not much different from normal people in terms of daily activities.

“As time passed, I have learned and understood all the conditions. Psychologically, I don’t feel myself different from other people. So far, it (the disease) is well controlled, no unexpected situation, just have to take medicines every day.” [A07]

Most participants regarded CML as a ‘chronic disease’ and hoped to maintain a stable control of the disease in the long term. They were satisfied with improvement in disease-related fatigue after receiving imatinib treatment and hence being able to maintain body functions and carry out daily activities (e.g. returning to work, sharing family child-care and housekeeping responsibilities) and relieve burden from carers.

“I exercise a lot and keep a normal health condition. My wife is at work, I am the ‘house husband’ and I have being keeping myself very busy. I just came to pick up my medicines this morning, but the nurse insisted that I need to have a regular check, that’s strange, I can’t see why it’s necessary.” [A15]

However, those who were seeking employment or holding future career plans still felt unproductive and oppressed as they were conscious of their limited life expectancy and the interruptions in their daily routine as a consequence of regular outpatient visits. One of the main concerns for participants was the disability and financial burden due to the deterioration of the disease.

“Our company is conducting several big projects overseas, such as the manufacture in Vietnam; I have to decline the project because I have my regular appointments.” [A06]

### Misconceptions about disease progression

The complete molecular response of CML treatment is defined as undetectable BCR-ABL transcripts, and the long-term therapeutic target for CML treatment is to maintain molecular response but only cytogenetic response. However, except for the physical and psychological discomforts, most participants were unclear about indicators of disease progression and therapeutic targets. Of the routine haematological tests, white blood cell count was found to be the most frequently referred parameter for disease status. Participants often regarded ‘increased complete blood cell counts’ as the proliferation of cancer cells and a metaphor for metastasis.

“It (the white cell) grows very quickly and the number multiplies in hundreds of thousands, if treatment can’t catch up (to kill cancer cells), then the quicker it grows, the faster the caner spreads.” [A09]

Some participants expressed that ‘no bad cell’ or ‘no Philadelphia chromosome’ represents a controlled disease condition after bone marrow biopsy. In addition, recurrent lesions gene (BCR-ABL transcript) tested by polymerase chain reaction (PCR) was seen as the sign of relapse by some participants, although the rising levels may merely indicate a loss of molecular response to treatment [33]. Even some participants who achieved complete molecular remission misunderstood that CML has an ‘incubation period’ and thought their condition was only temporarily under control.

“The doctor said my current condition is good because the molecular and haematology tests are beyond the scale. However, it doesn’t mean I have no ‘bad cell’, that’s the limitations of the tests. I did have a bone marrow test before, but not in the past two years. I hope I can have a bone marrow test, it’s more accurate.” [A11]

Bleeding was perceived to be the most commonly mentioned disease-related symptom, and participants generally avoided cuts and were cautious about wound-related bleeding and infections. Participants often had the misconception that blood loss via blood test or bleeding wounds might weaken their immune system, and increase susceptibility to ailments (such as the common cold). Being anxious about disease progression, participants were prone to react to the symptoms, which then led to frequent visits to emergency rooms or higher tier of medical facilities (e.g. medical centre).

“If I got a cut, I used to recover within a day or two, but since I took this medicine, oh my God even with a minor cut, I have to visit surgeon and get both pills and ointment! Sometimes, I get antibiotic injections, three continuous injections to get rid of the germs (to avoid cellulitis), I am so scared!” [A31]

### Factors associated with adherence to imatinib

Emerging themes from the interviews revealed patients’ beliefs on the efficacy of imatinib. Most participants experienced a rapid drop of white blood cell counts below the normal range after treatment, and believed in the efficacy of imatinib to control CML. In particular, those who received other therapies (e.g. interferon and chemotherapy) prior to imatinib regarded it superior to other treatments due to the advantages of low toxicity, mild adverse effects and oral route of administration. Therefore, most participants expressed that they would not stop, change or reduce the imatinib dose. Furthermore, participants suggested that they favoured long-term prescriptions for the maintenance of refills in order to avoid regular outpatient visits.

“I used to have interferon, but this (imatinib) tiny tablet is much better, as you can’t have interferon injections for ten years!… However, this long-term medicine is for chronic disease, a two-week schedule just passes too quickly, we should be allowed to have a long-term drug supply and only come to visit the doctor when we don’t feel right.” [A15]

Although participants were generally adherent to imatinib treatment, their concerns about the potential ‘resistance’ also influenced their medication behaviours. Participants were aware that long-term imatinib treatments could lead to resistance, but they had adopted the concept of anti-microbial resistance mechanism and believed that interrupting or changing medications would result in resistance to imatinib. In contrast to other chronic conditions, we found that participants seldom used traditional Chinese medicine or herbal medicine due to the concern that drug–drug or drug-food interactions might reduce the efficacy of imatinib.

“I feel resistance could happen after long-term use of drugs. I don’t have any medical concepts, but similar to ‘viruses’, virus resistance develops after long-term use of drugs, if we don’t take the drugs appropriately.” [A22]

### Concerns and management of adverse drug effects

Imatinib-related adverse effects were perceived to be the key concern of treatment. It was participants’ general view that the potential life-long treatment would lead to further harm to their health. Some participants even perceived the incidence of side effect as an indicator for disease progression. In addition, they often mistook the abnormal laboratory results or symptoms of imatinib-related adverse effects for occurrences of new diseases before the adverse effects were confirmed by physicians. Some participants complained that they lacked relevant information to monitor the adverse effects.

“The problems appeared one by one after different tests, and it was only until recently I realised they are the side effects of drugs.” [A05]

“…However, we worry the long-term use of Western medicine will damage liver or kidney, some doctors would test it (liver or kidney functions) but some won’t. How supposedly should we know whether to test it or not? But we definitely worry about it.” [A03]

Imatinib-related adverse effects were the most common reason for participants altering their treatment. To cope with the adverse effects, participants either reduced the dose of imatinib or adopted other approaches such as taking imatinib with or soon after a meal to reduce uncomfortable nausea or vomiting, or to take imatinib before bedtime rather than in the morning to avoid the uncomfortable vertigo (which often occurs half to two hours after imatinib intake).

“I changed to take the medicine before bed-time or after a meal. If I take it with an empty stomach, I will definitely vomit it out in ten minutes.” [A18]

In addition to imatinib-related adverse events, it was found that detrimental impacts associated with ‘polypharmacy’ for managing imatinib-related adverse events also worried participants. Most symptoms commonly raised by participants (e.g. oedema, nausea, vomiting, diarrhoea, insomnia, muscle pain, muscle cramps, poor appetite, itch and rash) are generally mild and can be managed by other oral medicines. However, some participants doubted whether long-term intake of the ‘rescue medicines’ for relieving the imatinib-related side effects was necessary, and worried about other possible adverse effects associated with taking too many rescue medicines, e.g. diuretics-related nocturnal frequency and nephrotoxicity, zolpidem-related sleepwalk and non-steroidal anti-inflammatory drugs related stomach upsets.

“The doctor prescribed a (diuretic) tablet once a day for me, but I only took half of it per day, because I worry long-term use of it will damage my kidney.” [A02]

## Discussion

This study found that participants who received routine imatinib treatment accepted CML as a chronic disease and were generally adherent to imatinib treatment. However, patients’ knowledge of their disease progression, therapeutic monitoring indicators, resistance to treatment and symptoms of side effects were not exactly correct. These perceptions increased patients’ concerns on disease progression and potentially incurred more medical resources, but the worries on poor treatment effects associated with resistance to imatinib also reinforced good adherence and beliefs on efficacy of imatinib. Concern on the adverse effects and associated polypharmacy was the main reason that patients altered imatinib therapy.

The patient journey during their treatment of CML presents a similar emotional pattern as manifested in other types of cancer treatment. Macdonald et al. [31] suggest a five-stage treatment journey for metastatic gastrointestinal stromal tumour patients which includes ‘crisis’ at diagnosis, ‘hope’ at initial treatment, ‘adoption’ at response and monitoring, ‘new normal’ under chronic management, and ‘uncertainty’ at resistance or progression stage. As this study recruited CML patients who routinely received imatinib treatment, it is not surprising to observe that they had accepted the chronicity nature of the disease and adapted to the treatment routine, two key stages constituting participants’ adherence to treatment.

Despite being adherent to the treatment, participants lacked sufficient knowledge of disease progression and therapeutic effects and hence were prone to misinterpreting laboratory results and judging the treatment outcome by the occurrence of symptoms, adverse effects and daily activities (function of life). This also indicates a communication gap between CML patients and healthcare professionals.

For example, PCR offered a parameter for monitoring long-term treatment response but the appearance of recurrent lesions gene in PCR does not necessarily mean that a relapse will occur. Resistance of imatinib is caused by gene mutation. Patients’ misconceptions about the condition of the disease might cause unnecessary anxiety, change medication-taking behaviours and even worsen adherence. A previous study has shown that establishing patient-physician trust and communication can have a positive impact on adherence [21].

Experiences of the treatment effects and knowledge of consequences caused by non-adherence to imatinib are important factors contributing to CML patients’ adherence to imatinib; and imatinib-related side effects and polypharmacy are the key concerns that may jeopardise adherence. Most findings from the present study are consistent with those from a previous study which was reported by Eliasson et al. [21] that took an in-depth approach to investigate the reasons for non-adherence to imatinib in a small target group of CML patients.

Eliasson et al. [21] enrolled 87 CML patients who had been prescribed imatinib for a minimum of 2 years at a teaching hospital in England, and monitored their imatinib taking for 3 months using a medication event monitoring system (MEMS, an electronic device fitted in the cap of the medication bottle to record the opening time and date), and 21 in-depth interviews were then conducted. Seventeen patients were categorised as non-adherent as their MEMS showed an adherence rate of less than 90 %; they generally regarded that missing several does will not damage the treatment efficacy. However, an adverse drug effect is still the main reason to non-adherence for long-term imatinib users.

A variety of methods have been used to measure adherence to imatinib in previous studies. For instance, the microelectronic monitoring systems [18], Basel’s assessment of adherence scale, pill counts, scheduled appointments ratio [19], prescribing interruption and medication possession ratio [20], and self-reported visual analogue scale [19] were referred to. However, due to the complexity of adherence, there is currently no gold standard to measure adherence to imatinib in clinical practice [1, 34] and no conclusive operational definition of good adherence [16]. Based on our findings, it is significant to consider and measure imatinib adherence in multiple domains such as medication behaviour (e.g. delay medication taking), administration routes (e.g. oral taken) and individual beliefs of disease and treatments.

In addition, previous studies have also showed that CML patients’ adherence to imatinib can also be influenced by increased concomitant medications and adverse effects [18, 20]. In the stage of severe CML (accelerate and blast phases), prescribing higher doses of imatinib would cause more adverse effects and poor adherence [35]. For patients with multiple co-morbidities, adherence might be reduced due to adverse effects, drug–drug interaction or polypharmacy. Previous research has suggested that finding ways to deal with side effects and using prompts as reminders to take the medicine are factors associated with good adherence to imatinib [21].

From the patients’ perspective, self-monitoring and self-managing symptoms of adverse effects were the easiest way to manage imatinib-related adverse effects [36]. Their experience also suggests that adopting an alternative medication-taking approach might relieve nausea, vomiting and vertigo and exercise can lessen oedema and muscle pain, thus reducing patients’ anxiety and avoiding the consequences of polypharmacy.

This study also suggests that the delivery of healthcare information and communication between patients and healthcare professionals need to be improved. Areas such as patients’ misconceptions about disease progression, monitoring effects from treatment and resistance of imatinib require further clarification, and healthcare professionals should improve patients’ knowledge on making appropriate judgment regarding effects from treatment, dealing with missing doses and adverse effects at the occasions of face-to-face patient counselling or medicine utilisation review, thus reinforcing the importance of adherence.

To the best of our knowledge, this is the first study using an in-depth qualitative approach to explore Taiwanese CML patients’ perceptions on imatinib treatment. This study recruited almost all active patients of the outpatient clinic and took a wider scope to explore patients’ treatment journey and experiences. The results inform the key issues of adherence to imatinib and reveal the information gap between patients and healthcare professionals.

However, the transferability of the results is uncertain as this study only involves CML patients who were regularly followed up in the hospital, and hence patients might experience better treatment effects and have better health awareness. The perceptions of those who were newly diagnosed with the disease or those suffering from poor treatment effects are still not clear. In addition, caution needs to be taken in interpreting the results and applying them to other countries, as this study was conducted in Taiwan where the National Health Insurance covers nearly 99 % of Taiwan’s population and patients are free to access different health care facilities, and hence affordability is less an issue as compared to adherence of imatinib.

## Conclusions

Taiwanese CML patients who are routinely followed up in hospital generally show good adherence to imatinib. However, patients’ misconceptions about disease progression, treatment side effects and concerns about adverse drug reactions can jeopardise their adherence to drug treatments. The provision of appropriate information and patient counselling services may improve the adherence to imatinib and patients’ outcomes, which are also possible avenues for further research.

## Appendix: Content of the interview schedule

- Could you tell me what do you know about CML?
- Could you tell me how your CML was treated?
  - Are there any treatment experiences that you would like to talk about?
- How do you feel about the treatments that you received?
  - How do you know the treatments are working?
- How do you take your medications?
- How do you feel about the drugs that you are having?
  - Are there any experiences about the drugs that would like to talk about?
- Is there anything else that you would like to talk about?
